# 

**Published:** 1864-06

**Authors:** 


### DENTAL REGISTER

### OF THE WEST.

EDITED BY

J. TAFT AND GEO. WATT.

JUNE,–1864.

---

**MONTHLY:**

**Three Dollars per Annum, in advance.**

---

CINCINNATI:

J. TAFT, PUBLISHER.

J. Taft, Dentist, 172 Race Street.

